# Health care providers’ perspectives regarding the use of chlorhexidine gel for cord care in neonates in rural Kenya: implications for scale-up

**DOI:** 10.1186/s12913-017-2262-8

**Published:** 2017-04-26

**Authors:** Angela Muriuki, Francis Obare, Bill Ayieko, Dennis Matanda, Kenneth Sisimwo, Brian Mdawida

**Affiliations:** 1 Save the Children, Matundu Close, Off School Lane, Westlands, P.O. Box 27679, Nairobi, 00506 Kenya; 2 Population Council, Avenue 5, Rose Avenue, P.O. Box 17643, Nairobi, 00500 Kenya

**Keywords:** Health care providers, Chlorhexidine, Cord care, Neonates, Rural Kenya

## Abstract

**Background:**

This paper explores the perspectives of health care providers regarding the use of 7.1% Chlorhexidine Digluconate (CHX) gel that releases 4% chlorhexidine for newborn umbilical cord care under a managed access program (MAP) implemented in Bungoma County of Kenya. Understanding the perspectives of providers regarding CHX is important since they play a key role in the health system and the fact that their views could be influenced by prior beliefs and inconsistent practices regarding umbilical cord care.

**Methods:**

Data are from in-depth interviews conducted between April and June 2016 with 39 service providers from 21 facilities that participated in the program. The data were transcribed, typed in Word and analyzed for content. Analysis entailed identifying recurring themes based on the interview guides.

**Results:**

Use of CHX gel for cord care in neonates was acceptable to the health care providers, with all of them supporting scaling up its use throughout the country. Their views were largely influenced by positive outcomes of the medication including fast healing of the cord as reported by mothers, minimal side effects, reduced newborn infections based on what their records showed and mothers’ reports, ease of use that made it simple for them to counsel mothers on how to apply it, positive feedback from mothers which demonstrated satisfaction with the medication, and general acceptance of the medication by the community. They further noted that successful scale-up of the medication required community sensitization, adequate follow-up mechanisms to ensure mothers use the medication correctly, addressing issues of staffing levels and staff training, developing guidelines and protocols for provision of the medication, adopting appropriate service delivery approaches to ensure all groups of mothers are reached, and ensuring constant supply of the medication.

**Conclusion:**

Use of CHX gel for cord care in neonates is likely to be acceptable to health care workers in settings with high prevalence of neonatal morbidity and mortality arising from cord infections. In scaling up the use of the medication in such settings, some of the health systems requirements for successful roll-out can be addressed by programs while others are likely to be a persistent challenge.

## Background

### Introduction

The health workforce is one of the key components of a well-functioning health system besides good leadership and governance, sound financing strategies, availability of essential medical supplies, innovative service delivery approaches, and proper health management information systems [1]. The ability of the health workforce to adequately respond to the needs of consumers of health services is, in turn, dependent on staffing levels, training, distribution, motivation, and retention [1–3]. However, the performance of adequate, well-trained, evenly distributed, and highly motivated health workforce may still be affected by other health systems factors such as leadership, governance, and supplies [2, 3]. Developing countries particularly face challenges with respect to ensuring efficiency in all the elements of a well-functioning health system [4, 5]. The challenges may be exacerbated in contexts of rapid changes in governance, financing, medical technologies and products, service delivery approaches, and information technology which may exert greater strain on the existing health systems of developing countries.

Guidance on appropriate newborn umbilical cord care is one area that has witnessed changes over time, which health systems of developing countries could experience challenges adjusting to. Evidence shows that there have been inconsistent practices using a variety of cleansing agents and techniques over time including alcohol-based solutions or antiseptics, antimicrobial treatment, and natural healing or dry umbilical cord care [6–8]. In addition, due to socio-economic and socio-cultural circumstances, different communities in low-resource settings apply unhygienic substances to the cord thereby increasing the chances of neonatal infections and mortality [9–12]. As a result, developing countries continue to bear the greatest burden of neonatal morbidity and mortality [13, 14]. Based on results from randomized clinical trials that showed that using 7.1% Chlorhexidine Digluconate (CHX) that releases 4% chlorhexidine for umbilical cord care significantly reduces neonatal morbidity and mortality in regions with high prevalence [15–19], the World Health Organization (WHO) included it in the essential medicines list for umbilical cord care in neonates in 2013 for use in settings with neonatal mortality rates of 30 deaths per 1000 live births or higher but also in regions with low neonatal mortality where the application of harmful traditional substances such as cow dung to the cord stump exist [20].

CHX is registered for topical use in Kenya but it is still not widely available. In order to generate evidence to inform the national roll-out of the medication, Save the Children began implementing a program in Bungoma County in western region of Kenya in early 2016 that involved availing 7.1% CHX in gel formulation to mothers through a controlled managed access program (MAP). The program was funded by GlaxoSmithKline (GSK) and implemented by Save the Children in collaboration with the Ministry of Health and the County Department of Health in 21 purposively identified public hospitals, health centers and dispensaries in Bungoma County while the evaluation was conducted by the Population Council. The introduction of the medication through health facilities was informed by a number of factors. First, since its use requires observance of some hygienic practices such as handwashing before application, the health facility was the first logical entry point where such hygiene could be observed and mothers could be educated on how to observe the same at home. Second, it was a more cost-effective way of reaching several mothers at the same time with information on the hygienic practices and application procedures as opposed to visiting each mother at home. This was later evident from interviews with mothers who used Chlorhexidine that showed that the approach was very effective in helping them understand how to apply it at home, including observing handwashing before and after application. Third, in such a rural community, most mothers trust information given by trained health care providers than anybody else. It was therefore anticipated that facility introduction would increase chances of correct use of the medication if the information came from trained health care providers.

This paper explores the perspectives of service providers regarding the use of CHX gel for newborn umbilical cord care under the program. Understanding the perspectives of service providers is, in turn, important for informing strategies for scaling up the use of the medication. This is critical given the key role the health workforce plays in the health system and the fact that their views could be influenced by prior beliefs and inconsistent practices regarding umbilical cord care. In addition, although there are no published studies that examined health care workers attitudes towards the use of Chlorhexidine for umbilical cord care, available evidence shows that the environment in which an intervention is introduced—including health systems and community context—may influence the acceptability of certain newborn care practices such as delayed bathing and dry cord care among mothers and health care workers, especially in rural settings of developing countries, thereby requiring negotiations for alternative practices [12, 21].

### Context

Until 2013, the national guidelines for Kenya recommended dry cord care but there is evidence that health care providers use alcohol-based solutions, methylated spirit or iodine to clean the cord [9]. In addition, given that a substantial proportion (38%) of births in the country occur at home or outside of health facilities [22], it is likely that many mothers use unhygienic substances for cord care. The proportion of births in Bungoma County that is delivered outside of health facilities (59%) is higher than the national average [22], suggesting that most infants in the county are exposed to unhygienic cord care practices. Available evidence shows that the infant mortality rate in the county is 65 deaths per 1000 live births compared with the national average of 39 deaths per 1000 live births while the neonatal mortality rate is 24 deaths per 1000 live births compared with the national average of 22 deaths per 1000 live births [22, 23]. In addition, the county has the highest fertility rate (of five children per woman) and the lowest proportion of births occurring in health facilities (41%) among the four counties (Bungoma, Busia, Kakamega and Vihiga) in western region of Kenya [22]. Experience working in the study setting further showed that practices involving application of harmful traditional substances to the cord stump exist, which justifies the use of Chlorhexidine for cord care despite the neonatal mortality rate being below the threshold recommended by WHO. In particular, interviews with mothers who used Chlorhexidine showed that the application of traditional substances such as cow dung, traditional herbs, soil, soot, feces of lizard, feces of bat and chicken droppings to the cord stump still exist in the community.

It is against the backdrop of poor maternal, newborn and child health indicators that the CHX MAP was implemented in the county. Implementation of the program began in February 2016 and involved training of health care providers from the participating facilities on dispensing the medication and reporting adverse events; developing and disseminating information, education and communication (IEC) materials on CHX; and supplying the medication to the facilities. Although the program was facility-based, community midwives, community health extension workers (CHEWs), community health volunteers (CHVs), and birth companions (former traditional birth attendants attached to facilities to accompany mothers for health services) from the county were sensitized about the medication so that they could disseminate the information in the community and encourage mothers who gave birth at home to visit the facilities for CHX. Each mother was provided with a packet of CHX that contained seven sachets. The first application was done by the provider at the facility as per the guidelines and mothers were shown how to apply the rest at home. Mothers were then instructed to use one sachet once a day for the remaining six days and discard whatever remained in the toilet. However, CHX was applied only once at the facility for low birthweight babies (less than two kilograms). This was a policy decision made during the design of the intervention based on reports of increased risk of CHX irritation in low birthweight or pre-term babies and the fact that the most efficacious dose of CHX is the first dose given at or as close to the time of cutting of the cord as possible. However, for mothers who gave birth at home and were referred to health facilities for CHX by CHVs and birth companions, the efficacy of the medication might have been reduced due to delayed initiation of the first dose.

## Methods

Data are from a cross-sectional study that was conducted between April and June 2016 involving in-depth interviews with 39 service providers (25 female and 14 male) from all the 21 facilities participating in the CHX MAP. Two providers were targeted in each facility although only one provider was interviewed in a few of the facilities that did not have adequate staff. All service providers interviewed were nurses, which is the cadre of health care staff that mostly interacts with patients. Verbal permission was first obtained from the manager of each facility to conduct the interviews with the providers. Providers were then purposively identified based on whether they participated in the CHX MAP and were available and willing to participate in the interviews.

The interviews were conducted in English by research assistants with training in qualitative data collection. An interview a guide was used to capture information on the acceptability of using CHX for cord care among providers, their attitudes towards the medication, the health systems requirements for providing the medication, their experiences offering CHX to mothers of newborns, and their views regarding the IEC materials on CHX. The interviews were scheduled when providers were free to avoid interfering with normal service delivery at health facilities and were audio-recorded with consent of participants. The data were transcribed, typed in Word and analyzed for content after all the interviews were done. Analysis involved reading all the transcripts and identifying recurring themes from providers’ responses based on the interview guides.

Written informed consent was obtained from all participants before conducting the interviews. The study obtained ethical approval from the Institutional Review Board of the Population Council (Protocol 729) and the Ethics and Research Committee of Kenyatta National Hospital/University of Nairobi (P53/02/2016) while the National Commission for Science, Technology and Innovation (NACOSTI) granted the research permit (NACOSTI/P16/8900/10383).

## Results

### Existing cord care practices

Providers reported fairly standard approaches for cutting the cord for births occurring at the facilities including measuring the cord (about 2.5 cm) from the base, clamping using cord clips, and cutting using sterile scissors. Some providers reported that they tie the cord using a thread when they run out cord clips and that they ask mothers to buy razor blades for cutting the cord when there are no sterile instruments. With respect to substance application, providers noted that the practices varied over time and involved using methylated spirit, normal saline or dry cord care. One provider indicated that such changes created confusion for mothers and contradictions among providers. Cord stumps that drop off while mothers are still at the facility are either disposed of in a pit latrine or incinerated.

*For taking care of the cord, we have been using spirit, methylated spirit. At one time they said no, we can’t use spirit; we should use normal saline. Then we did with normal saline. Then it came to a point they said no, we leave it dry; we don’t apply anything. Then later on they said no, instead of leaving it dry, let the mothers just use water.* (Female nurse, 31 years work experience)

*In most cases like the normal saline and spirit we used to use, it used to work but the problem was the facility used to be affected in this way because once the mother goes home there is no way we shall give that normal saline to carry with her home and use it. So, we used to encourage them to use what would be locally available—that they could use water added in some salt which is not measurable. You see, now we were contradicting [ourselves]. We could start well but once the mother delivers and she goes home within 24 h, now what will happen after there, it could not be determined.* (Female nurse, 10 years work experience)

Cord care practices in the community for births occurring at home had serious challenges. Providers reported that strings from piece of cloth, pullover, blanket, banana leaf sheath, corn husks or sisal are used to tie the cord. The cord is then cut using a razor blade, a knife, finger nails, or sugarcane peels. The substances that are applied to the cord after cutting include cow dung, traditional herbs, ash, soot, baby powder, spirit (whether surgical or methylated), and droppings of chicken, lizard or bat. The practices often resulted in umbilical infections and sometimes death. Providers attributed these practices to socio-cultural beliefs, lack of information on available services, inability to afford the costs of health care, long distances to health facilities and lack of birth preparedness.

*The common one [instrument for cutting the cord] in the community is just a razor blade…or they could even use a knife and…there are traditional birth attendants who may also use a [finger] nail.* (Female nurse, 31 years work experience)

*Some of them use the droppings of a bat, they apply on top, the place where they have already cut…they say it is medicinal, it [the cord] shrinks and dries faster. Then some of them say they buy powder, they use it on top. Then others buy…the spirit, they don’t know which spirit, they buy any spirit whether methylated or surgical and they apply around there.* (Female nurse, 5 years work experience)

*The worst case scenario is there is a day I had a mother with a three-week old baby, with maggots…Yeah, the mother is comfortable, the child is crying, fever set in. So what I did is, I just removed those maggots, cleaned the cord. I had to look for the CHV [community health volunteer] who lived near that mother so that she could daily remind that mother to come for dressing.* (Female nurse, 10 years work experience)

### Chlorhexidine gel has done it

One provider summarized his views regarding the use of CHX gel for cord care in neonates by stating that ‘CHX has done it’. All providers interviewed expressed similar sentiments for various reasons. In particular, some providers mentioned that CHX is easy to use, which makes it easy to explain to mothers and for them to use it thereby simplifying counseling on and process of newborn care. Others reported that CHX makes the cord heal fast based on feedback they got from mothers. Some indicated that facility records and reports from mothers showed that CHX reduced newborn infections even within the short time it was in use while other providers reported that it had minimal side effects. Another provider alluded to indirect health benefits of using CHX for the baby in that the mother will be psychologically happy, which in turn stimulates the production of enough breast milk for the baby.

*To start with, CHX has done it. Why should I say it has done it compared to the other methods and systems that we used to advise our mothers? [It] is because CHX does not require complicated knowledge to use; it is just a matter of updating the mother showing them how it is being applied and then somebody picks it from there. Secondly, CHX reduces the span of healing or falling of that stump…you see the span of time taken is short. Another thing that CHX has done compared to the other methods that we used to have is that it saves time because it is being applied once per day, so it saves time for the mother and for the family members. And…even the father can take care because it is applied only once; it has no burning sensation compared to spirit that was previously used.* (Male nurse, 8 years work experience)

*Well, I can say I thank those who brought CHX because though saline was working, yes, but the chances of harboring infections was high, because maybe the mother could use contaminated cotton or contaminated kind of cloth, you know, to do the cleaning of the cord, and this was bringing more infection. But for CHX before they apply, we normally tell them to wash their hands, we instruct them very well, yeah, they use their finger to apply. They are not supposed to use a cloth or what, a finger is just enough to apply, yeah…The other advantage of CHX, it is the simplest and the safest way…it is not time consuming.* (Female nurse, 6 years work experience)

*Before introduction of CHX, we have been having cord sepsis, many of them and I can proudly say [that] though it [provision of CHX] has not been in practice for long, for the past two to three months we have not got more than two cases of cord sepsis because I am the one who makes reports, I go through out-patient records, I go through all the records while compiling reports, we don’t get sepsis.*(Male nurse, 4 years work experience)

*For us, it has lessened microteaching like we would say “mummy, now we have said that if you go home, don’t use lizard’s dropping, don’t use cow dung, don’t use this.” But right now we just tell the mother that to care for the cord we have this right gel. Now if you go back home please, I don’t want you to go back to traditional ways of caring for the cord. So it has just lessened our work of explaining to the mother.* (Male nurse, 13 years work experience)

### Factors facilitating provision of chlorhexidine gel

Interviews with providers showed that the factors that facilitated the provision of CHX were both supply- and demand-related. The supply-side factors included absence of or minimal infrastructural changes required to provide the medication, continuous availability of the medication (no stock-outs), provider training that was conducted before roll-out, positive attitude of health care staff towards the medication, and availability of informational materials. Most providers interviewed indicated that they did not require any changes in infrastructure or personnel to provide the medication although a few facilities made some changes. Such changes included ensuring that cabinets for storing the medication were available in the pharmacy, maternal and child health (MCH) and child welfare clinics (CWC) to enable mothers obtain the medication from any of these units. Facilities also received a separate register for recording mothers who were given CHX since the existing maternity registers did not have provision for it.

Providers further appreciated the support they received from the implementers of the program with respect to ensuring that they had continuous supply of the medication. They also considered the training they received before roll-out as important not only for helping them understand the medication and how it works but also enabling them develop positive attitudes towards it. The availability of posters on CHX, which were developed as part of the program, reminded providers of how to apply the medication during counseling and demonstration for mothers.

*One, in our delivery room, we have a tray that has all the commodities needed for the baby…So our addition was to include CHX. We make sure we have included a CHX box there and a list where to record, so that is one of the changes. Two, we have made sure we hang a poster…it is over there, there is a couch where we receive the baby. If there is resuscitation we do it there, directly at your eye level you will see the IEC material to show application of CHX. So any time you are working on the baby there, you look up and you will see a chart to show you how to apply CHX, so you will always remember to apply CHX. And even in our comments in the maternity register, when we began we had not included it but with time we have started including it…We have decided to do that to remind ourselves and make sure we have given it to every newborn. Again we have improved on screening of mothers who bring newborns delivered at home in our CWC [child welfare clinic], yes, to ensure that we get them. Whoever we get as we are providing other postnatal [care] services we also remember to give CHX for the baby.* (Male nurse, 4 years work experience)

*Because of the continuous supply, we have not run out of them [CHX], so we have not stopped giving CHX to our mothers to apply to their babies. So, it has actually been very possible and again at the same time like for me I am working in NBU [New Born Unit], any time in the morning when you go asking which mother has not received it, is like they already know. Maybe a mother who delivered at night was missed. She will carry up the hand, “I have not had.” So they are really optimistic they must apply [CHX].* (Female nurse, 31 years work experience)

*So when it was introduced in the facility, we had a CME (Continuous Medical Education). All the staff were involved, that is, the nurses and the clinical officers, they trained us on how to apply. Once the baby is born, you make sure the baby is given CHX before discharge…Then our CHVs [community health volunteers] were informed, so…they went to their various CUs [community units], they also talked to those mothers.* (Female nurse, 2 years work experience)

From the demand-side, ease of use of CHX, acceptance of the medication by the community, and positive feedback from mothers who used the medication facilitated the provision of the medication. As already noted, ease of use of the medication simplified the counseling process for providers. In addition, acceptance of the medication by the community made the work of providers easy, with one of them pointing out that the positive feedback they received from mothers who had used the medication was enough motivation to continue offering CHX. The positive feedback from mothers was largely because of fast healing of the cord, reduced costs of newborn care since the medication was free and mothers did not have to frequently buy soap for washing napkins soiled by the cord, absence of side effects and the fact that using the medication does not require high levels of education.

*In CWC [child welfare clinic] when you meet mothers, sometimes, I have heard testimonies from mothers. They believe this medication treats the cord and it has helped a lot. That is what those who come to CWC tell me, those who have used it. They say, “Daktari [Doctor], this medication is very good, since I started applying it on baby’s cord, the cord has healed so fast.” That is what they say. Those are some of the positive things they have said.* (Male nurse, 4 years work experience)

*Very simple reaction [from mothers]; it has saved them from other costs because we are still providing it free. It has also saved them from the cost of buying even bar soap [for washing napkins]. The other reaction they say it has no complications; it doesn’t require high levels of education; you are only shown and you go and do it.* (Male nurse, 8 years work experience)

### Challenges to provision of chlorhexidine gel

Similar to factors that facilitated provision of CHX, the findings showed that challenges to provision of the medication were both supply- and demand-related. The supply-related challenges included staffing and associated workload as well as counseling on and addressing side effects of the medication. Challenges of staffing and workload could limit ability of providers to reach all mothers with CHX especially when a few providers have to serve many clients with various health needs. One provider noted that although CHX was an additional workload in the care of newborns, it was their responsibility to offer it and it was the right of the babies to receive it. Another provider reported that some of them were apprehensive about counseling mothers on irritation if CHX gets to the skin of the baby and feared that it might cause rejection of the medication. A few providers reported not knowing what to do in cases of delayed cord separation or when mothers who delivered at home presented at the facility asking for CHX when the time frame for using it had elapsed.

*Yes, there are challenges. Sometimes I fear that we may be missing out on mothers who deliver at home and come to CWC [child welfare clinic] the following day and maybe because of so much workload…Sometimes you come on Thursday, you get more than fifty mothers have come for clinics, your colleague is in training, you are all alone in maternity, in CWC, you are in outpatient, you are in family planning, you slept at work at night. You can remember to give CHX but you may miss out on a mother who has brought a baby at CWC, yes. So, there is a challenge regarding staffing and that may affect provision.* (Male nurse, 4 years work experience)

*Another challenge, to be specific, that I underwent is after this child was born here in the facility and then we gave CHX and then after this, the mother administered the CHX. But unfortunately the cord did not fall off. So it was a challenge until it left me in a situation that I was forced to refer to a pediatrician who at least could be able to know why.* (Male nurse, 26 years work experience)

*Yes, it is workload but we are trying to fit it in our procedures because Chlorhexidine has now been put for our mothers and children to use, so it is our work to do it. It is an additional workload but we have tried to fit it in because also on the part of the baby…one, we have vitamin K. If it is there, you have to give. We have TEO [tetracycline ointment for the eyes]; that one we are also supposed to give immediately after delivering the baby. Now it [CHX] has been added and it is the right of that baby, so if you don’t give it, you will ask, “What have I done to that baby?”* (Female nurse, 42 years work experience)

The demand-related challenges, on the other hand, included inability to follow up on mothers to ensure that they used the medication correctly and the requirement for washing hands before applying the medication. Interviews with providers showed that mothers who were given CHX but went back to facilities with complaints of delayed healing of the cord had not used the medication they were given, used it with other substances, or did not follow advice on cord care including not covering the cord with diapers. One provider also mentioned that some mothers experienced challenges with the requirement of washing hands before applying the medication while another pointed out the potential for misuse, especially for treating other wounds given that mothers considered the quantity in one sachet more than enough for a single application.

*I can remember there was a mother who came, it was around the tenth day and she had the CHX but the cord had sepsis. So I just came and interviewed her and I was like, “Did you really use the medication the right way as you were told?” She tried to hide and she told me she used CHX and it was over. In fact, the CHV [community health volunteer] from that village is the one who brought her, so she is the one who disclosed the truth and told me that the mother was not using CHX.* (Female nurse, 6 years work experience)

*She gave birth in our facility, so when the baby was having some problem with the abdomen she went to a different facility and they were like “maybe where you delivered they cut the cord wrongly that is why it is like that.” We counseled the mother; we realized the mother had a different problem altogether because we always tell the mothers…don’t cover the cord [with diapers] because wetness will spread and then infection can spread. But so far we have not had any infection apart from that one who came and the cord had not dropped off after three weeks.* (Female nurse, 31 years work experience)

*From the history, this other mother who came with sepsis said she was following the instructions…So, we tried to explore how they are caring for the baby generally and also how they are caring for that cord. But now it was like she was trying to hide some information. She was not applying CHX alone, it was like she was adding powder.* (Female nurse, 10+ years work experience)

*It becomes like a disadvantage but always applying and washing hands. “Daktari [Doctor], washing hands is giving us problem…always washing hands and packing”….A few ask why it can’t be in a powder form if it is possible to just sprinkle to save them from washing hands.* (Male nurse, 8 years work experience)

### Implications for scale-up

In spite of the reported challenges, all providers interviewed noted that the use of CHX for cord care should be scaled up nationally. They, however, pointed out that a number of supply- and demand-related activities should be carried out before scale-up. One such activity is community sensitization through mass media (television, radio, and newspapers), Chief’s *barazas* (meetings), road shows, churches and mothers who have used the medication in order to ensure acceptance. Providers also cited training of health care workers and community health volunteers as well as developing guidelines and protocols for procuring the medication as key steps before rolling out the program. Some providers also pointed out that the medication should be provided free of charge to avoid locking out mothers who cannot afford and who might resort to traditional methods for cord care. They further mentioned the need to ensure that there is no stock-out of the medication once it is rolled out.

*I think just the way we go with polio when we want to start something is that campaign through the media, something like that should be done through the media; television, radio, pamphlets all over so that the mothers know there is something new coming up and for them who are using they will be able to encourage others, “yes, I used this it is workable and it is good”…And maybe Chief’s baraza [meeting], if any, or now we are having the counties they are having their own assembly, they can still pass the same information. Or even in churches, and then CHVs, Community Health Volunteers who can go out, they can still spread the same [message] so that the uptake can go up and even higher.* (Female nurse, 31 years work experience)

*One, for a program to be successful, I feel that the community should be involved in every step, we train the health care workers, we train the Community Health Volunteers, we go down we have forums in the community, we tell them we are rolling out this, the community accepts it before it is done. If we don’t do that it will not work well. Two, we ensure steady supply, anything to do with stock-outs should end. If you start a program and there are stock-outs, it brings challenges.* (Male nurse, 4 years work experience)

*Maybe we put across a clear protocol on how to order them, how to receive them so that we know if the stock we have is finished, how do we get more stock, yeah, that process of ordering, procurement process should be clear, put in place and known everywhere in the facilities so that we don’t run out of them because sometimes we can run out of stock and you wonder now whom do you contact next.* (Female nurse, more than 10 years work experience)

*They should not sell [CHX]. You see, if they just put money…charges…mothers will do away with it…There are some mothers, they are very poor, they cannot afford.* (Female nurse, 3 years work experience)

## Discussion

Qualitative studies in Asia and sub-Saharan Africa showed culturally diverse practices and beliefs around umbilical cord care that suggested the need for evidence-based interventions such as the use of CHX to improve neonatal outcomes [24–27]. A key finding of this paper is that use of CHX for cord care in neonates was acceptable to health care providers in the facilities that participated in the program, with all of them supporting scaling up the use of the medication throughout the country. Their views were largely influenced by positive outcomes of the medication including fast healing of the cord as reported by mothers, minimal side effects, reduced newborn infections based on what their records showed and mothers’ reports, ease of use that made it simple for them to counsel mothers on how to apply it, positive feedback from mothers which demonstrated satisfaction with the medication, and general acceptance of the medication by the community. In view of previously inconsistent umbilical cord care practices [6–9] that some of the providers in the study considered confusing and contradictory, coupled with unhygienic practices in the community (which are influenced by socio-cultural, socio-economic and structural circumstances), the findings suggest that scaling up the use of CHX could simplify rather than complicate newborn care for health service providers and mothers alike. This is particularly the case given the evidence that even where providers recommend certain newborn care practices such as delayed bathing and dry cord care among rural communities, mothers still resort to alternative practices such as early bathing and applying substances to the cord due to certain cultural beliefs surrounding birth [12, 21].

Although health care providers in the study supported scaling up CHX use, they noted that successful scale-up hinges on addressing demand and supply issues. These include conducting community sensitization, putting in place adequate follow-up mechanisms to ensure mothers use the medication correctly, addressing issues of staffing levels and staff training, developing guidelines and protocols for provision of the medication, adopting appropriate service delivery approaches that do not lock out certain groups of mothers, and ensuring constant supply of the medication. Some of the issues were addressed by the program before or during implementation and can be addressed by similar programs during scale-up. These include community sensitization through CHVs, staff training, providing CHX for free and ensuring constant supply of the medication. However, ensuring adequate health workforce has remained a major challenge for health systems of many developing countries such as Kenya [3] and is likely to continue if the use of CHX for cord care is scaled up. In addition, in light of available evidence from randomized controlled trials showing no difference in neonatal mortality between CHX use and dry cord care among facility-based births in some settings [17, 28, 29], the findings of this paper suggest that scaling up the use of CHX should take into account the community context. In particular, the findings indicate that scaling up the use of CHX would be beneficial in settings where application of harmful traditional substances still exist and where mothers are likely to resort to such practices in the absence of acceptable alternatives due to cultural beliefs about birth and newborn care.

Furthermore, most developing countries rely on donor funding for supply of health commodities [30]. The availability of such funding might affect the supply of CHX and the service delivery approach that is adopted during scale-up. The service delivery approach might also be influenced by users’ willingness to pay for the medication. Studies, however, show that CHX is available at relatively low prices (0.01 US dollars in raw materials per baby) and that majority of users were willing to pay some amount and could borrow money if necessary [31, 32]. Whereas health care infrastructure is also a major challenge for developing countries [4, 5], the fact that providing CHX required no or minimal changes in facility infrastructure suggests that it might not affect scale-up. In addition, CHX has other advantages that may influence its provision such as long shelf life and non-requirement of cold chain for supplies [32].

The findings of the paper might be influenced by certain limitations. First, the paper is based on qualitative interviews with providers in public health facilities that participated in the program. Their views might not therefore reflect those of providers in private facilities. Second, although all facilities that participated in the program were included in the study, participants were purposively identified from each facility. It could be that the purposive identification of participants led to selection bias with only those with positive views being included in the study. However, the findings of the paper show that there were providers who reported experiencing challenges regarding counseling mothers on side effects of CHX while others mentioned encountering mothers who reported some challenges with using the medication. This suggests that the purposive selection of participants did not bias the findings towards positive views only. The interviewers did not also explore in detail whether the challenge with washing hands was socio-cultural, socio-economic or structural, which could inform the design of appropriate programs to address it.

## Conclusion

The findings of the paper suggest that the use of CHX gel for cord care in neonates is likely to be acceptable to health care workers in settings with high prevalence of neonatal morbidity and mortality arising from cord infections. In scaling up the use of the medication in such settings, some of the health systems requirements for successful roll-out (such as community sensitization, staff training and commodity supply) can be addressed by programs while others—such as staffing levels—are likely to be a persistent challenge.

## Abbreviations

CHEW: Community Health Extension Worker
CHV: Community Health Volunteer
CHX: Chlorhexidine
CME: Continuous Medical Education
CWC: Child Welfare Clinic
GSK: GlaxoSmithKline
IEC: Information, Education and Communication
KNBS: Kenya National Bureau of Statistics
MAP: Managed access program
MCH: Maternal and child health
NBU: Newborn unit
TEO: Tetracycline ointment
WHO: World Health Organization
